# Haemophilic Pelvic Pseudotumour: A New Surgical Option

**DOI:** 10.3390/healthcare9101269

**Published:** 2021-09-26

**Authors:** Gianluigi Pasta, Roberta Ruggieri, Salvatore Annunziata, Alessandro Gallese, Vincenzo Pio Gagliardi, Fabrizio Cuzzocrea, Matteo Ghiara, Mariaconcetta Russo, Paola Stefania Preti, Roberto Mario Santi, Mario Mosconi, Francesco Benazzo

**Affiliations:** 1 Orthopedics and Traumatology Clinic, IRCCS Policlinico San Matteo Foundation, 27100 Pavia, Italy; gianluigipasta@yahoo.it (G.P.); robertaruggieri9@gmail.com (R.R.); alessandro.gallese1@gmail.com (A.G.); vincenzopio.gagliardi01@universitadipavia.it (V.P.G.); cuzzofabri@gmail.com (F.C.); m.ghiara@smatteo.pv.it (M.G.); mario.mosconi@unipv.it (M.M.); fbenazzo@unipv.it (F.B.); 2 Department of Internal Medicine and Therapeutics—Clinica Medica II, IRCCS Policlinico San Matteo Foundation, 27100 Pavia, Italy; ma.russo@smatteo.pv.it (M.R.); paolastefania.preti@unipv.it (P.S.P.); 3 Thrombosis and Haemostasis Center, Azienda Ospedaliera Nazionale SS. Antonio e Biagio e Cesare Arrigo, 15121 Alessandria, Italy; rsanti@ospedale.al.it

**Keywords:** haemophilia, pseudotumour, pelvis, perioperative management, surgical treatment, complication

## Abstract

Background: Haemophilia is an inherited coagulopathy caused by the absence or dysfunction of clotting factor VIII or IX. Clinical manifestations are generally secondary to recurrent bleeding episodes mainly in the musculoskeletal system. Bleeding symptoms appear early in life and, when the disease is severe (when plasma factor VIII or IX activity is <1% of normal), joint and muscle bleeding may occur spontaneously. A pseudotumour is a recurrent, chronic, encapsulated, slowly expanding, muscle hematoma. Haemophilic pseudotumour is a rare complication of haemophilia which occurs, as a condition either from repeated spontaneous bleeding or coming from a traumatic origin, in 1–2% of haemophilic patients. Case report: A 32-year-old man with severe haemophilia A referred to our Clinic with a massive right iliac wing pseudotumour complicated by Staphylococcus aureus superinfection and skin fistulisation. In this report we describe the medical management and surgical treatment by the adoption of a novel surgical technique which involves the use of a pedicle-screw and rod system (PSRS), a polyglycolic acid MESH and bone cement in order to build up an artificial ilium-like bony mass. This case report highlights the importance of interdisciplinary approach and the efficacy of eradicating surgery as treatment, especially in the case of large and long-lasting lesions.

## 1. Introduction

Haemophilia is a recessive X-linked inherited coagulopathy caused by the absence or dysfunction of clotting factor VIII, for haemophilia A, and factor IX, for haemophilia B. This bleeding disorder has an incidence of 1:5000/10,000 male births [1] and its clinical severity is commonly related to the deficient clotting factor plasma level. Patients are classified as having mild, moderate or severe haemophilia depending on the level of the deficient factor, which can be >5% of normal in mild cases and <1% of normal in severe haemophilia; this is reflected in the frequency and causes of bleeding [2].

Clinical manifestations are generally secondary to recurrent bleeding episodes mainly in the musculoskeletal system where approximately 80% of all haemorrhages occur, usually starting from childhood [2].

The most common pathophysiological process reported in haemophilic patients is joint destruction caused by intra-articular bleeding, condition known as haemophilic arthropathy. Intramuscular haemorrhage is the second most common site of bleeding in hemophiliacs.

Pseudotumour is a rare complication of haemophilia which occurs, as a condition either from repeated spontaneous bleeding or coming from a traumatic origin, in 1–2% of haemophilic patients [3].

A pseudotumour is a recurrent, chronic, encapsulated, slowly expanding, muscle hematoma [2,3]. This leads to the formation of capsulated masses containing coagulated blood and necrotic tissue. Haemophilic pseudotumour was first described by Starker in 1918, the term “pseudotumour” derives from the roentgenographic skeletal changes consisting of areas of bone destruction and new bone formation as well as calcification and/or ossification of surrounding soft tissues, resembling a malignant skeletal tumour [3,4,5].

Actually, there is no consensus in terms of therapeutic management which in turn depends on variables such as pseudotumour location of the pseudotumour, its size and the characteristics of the patient.

We report a successful resection of a massive right iliac wing pseudotumours in a 32 years-old patient affected by severe haemophilia A treated with a novel surgical technique.

## 2. Case Presentation

### 2.1. Clinical History and Instrumental Assessment

Our patient was a 32-year-old man, coming from a country with low availability of factor replacement therapy, height 185 cm, weight 90 kg, professional driver, with severe haemophilia A (F VIII < 1%). Family history positive for severe haemophilia A (his aunt was affected). The diagnosis of severe haemophilia A occurred in his hometown during childhood because he was involved in several atraumatic intra-articular haemorrhages in the elbows, ankles and knees.

In March 2017, after an accidental fall skiing, he reported a blunt trauma of the right iliac wing with development of blood collection in that site.

**Figure 1:**
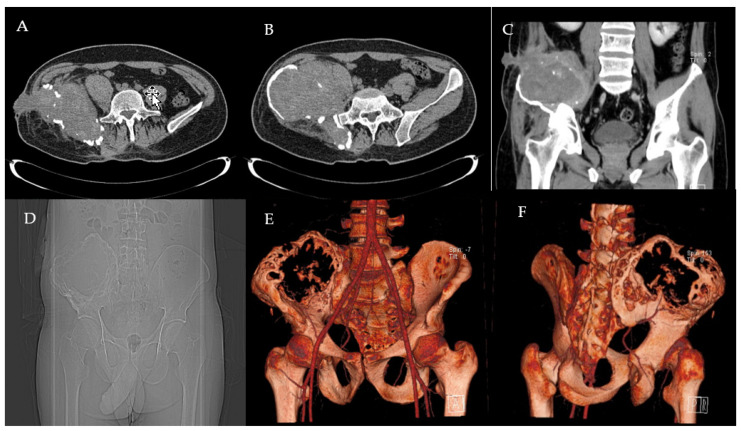
Preoperative CT scans (**A**–**C**), radiograph (**D**) and 3D reconstruction (**E**,**F**) of the pelvis with the right iliac pseudotumour.

Pelvic X-rays and bone biopsy were performed at the local hospital and osteosarcoma was excluded. In July 2017 he went to another Haemophilia Centre for the appearance of swelling, fistulisation, leakage of serum blood material and functional limitation of the right hip. He also reported an evening fever of >39°. The physical examination also revealed severe atraumatic functional limitation of the left elbow and the right ankle. Laboratory tests performed showed F VIII <1%, F VIII inhibitors: 0 BU/mL. A pelvis CT was performed, showing the presence of an abscess and a fistula at the right iliac crest (Figure 1). Staphylococcus aureus was isolated from the culture swab, therefore antibiotic therapy with Teicoplanin + Rifampicin was introduced but subsequently suspended for gastrointestinal intolerance. The patient started prophylaxis with pd-FVIII 30 IU/Kg three times a week. In June 2018, the patient was referred to our Clinic for right hip swelling, fistulisation, serum secretion, pain and functional limitation. Blood exams at the entrance showed Hb 8.6 g/dL, aPTT 40 s, F VIII 20.7% (range 70–120%), inhibitors F VIII 0 BU/mL, PCR 4.4 mg/dL (<0.5). A contrast medium pelvic CT scan demonstrated the presence, at the right iliac wing, of a large (10.1 × 14.2 × 10.7 cm) osteolytic lesion, inhomogeneous, septate and bounded by a peripheral wall of irregular thickness (6–7 mm). The lesion interrupted the cortical bone on both the posterior and anterior side of the iliac bone, also involving the sacroiliac joint plane and the sacral joint surface at S1-level. The lesion infiltrated both the gluteal muscle lodge posteriorly and the deep plane of the ileo-psoas muscle anteriorly. In addition, the presence of two subcutaneous fistulous communications was demonstrated, reaching the cutaneous plane respectively along the lateral side of the right flank and in the right paravertebral posterior lumbar region, at L5-level. Considering the patient’s medical history of haemophilia, a diagnosis of pseudotumour of the right iliac wing with an additional overlapping inflammatory-infectious complication was made.

Surgical treatment to remove the pseudotumour was proposed and accepted by the patient.

Pd-FVIII 20 IU/Kg were administered pre-operatively as a slow intravenous bolus every 12 h for seven days before surgery.

### 2.2. Surgery

**Figure 2:**
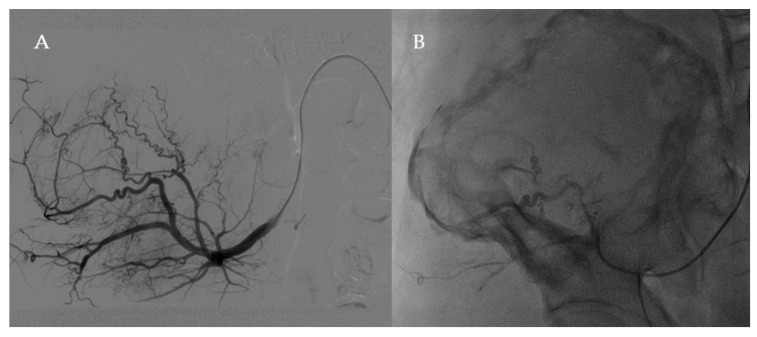
Angiography of arterial embolisations, before (**A**) and after (**B**) the procedure.

The day of the surgery, the patient underwent selective embolisation with Embosphere^®^ 500–700 of the right superficial gluteal artery and the right lumbar artery (Figure 2).

Just before the anaesthesia, pd-FVIII 50 IU/Kg was administered as a slow intravenous bolus. After that, 1 g of vancomycin was administered intravenously as intra-operative antibiotic prophylaxis. Under general anaesthesia, the patient has been placed in the lateral decubitus position. Support placed under the thorax allowed to put the patient’s pelvic area into a three-quarter anterior and posterior pelvic tilt during the intervention. Anterolateral and posterior access to the pelvis were used. The incision started along the contours of the iliac crest in a curvilinear manner and ends in the back with a vertical portion on the sacroiliac joint. The wing of the ilium and the sacrum are exposed, allowing for safety margins around the pseudotumour, which extend into the soft tissue. The iliac muscle was sectioned and detached from the endopelvic side of the ilium. The iliac vessels, ureter, lumbosacral trunk, sacral roots and sciatic nerve were identified and protected. On the exopelvic side, the gluteus muscles were moved as a unit with their fascia and skin, creating a lower myocutaneous flap.

After a layered dissection of the soft tissues, we reached the pseudotumour which appeared as a voluminous brownish-red mass with a pasty-like consistency; its capsule was emptied and removed. Subsequently, the right iliac crest was custom-fit by means of a pedicle-screw and rod system (PSRS), a metal bar and two fixing screws (one in correspondence with the acetabulum and the other on the sacro-iliac synchondrosis). Dead space between the implants and the remaining bone were therefore filled by antibiotic coated bone cement with the aid of two polyglycolic acid MESH in order to build up an artificial ilium-like bony mass, as well as graft compression.

Finally, two treble hooks were positioned for repositioning the pelvic muscles. One surgical drain was placed at the end of the surgery. Intraoperatively, a biopsy of the lesion demonstrated the presence of fibrin-haematic organised material with fragments of necro-inflammatory material and scattered microcalcifications. Swabs in fistulous passages were also performed and they showed that there was no superinfection. The patient was transfused intraoperatively with one unit of RBC

### 2.3. Postoperative Period

**Figure 3:**
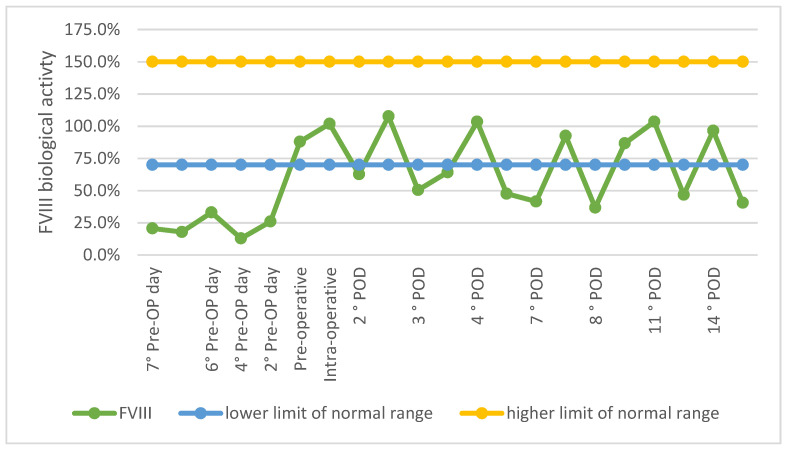
First hospital stay FVIII biological activity levels preoperatively, intraoperatively and post-operatively. Pre-OP: pre-operative; POD: post-operative day.

At the end of the surgical procedure, pd-FVIII 20 IU/Kg was administered as bolus, then pd-FVIII 20 IU/kg every 12 h on the first postoperative day; from the second post-operative day pd-FVIII 20 UI/Kg at 8 a.m. and 10 UI/Kg at 8 p.m., up to the 4th day postoperatively; from the fifth post-operative day pd-FVIII 30 IU/Kg at 8 a.m. and 10 IU/Kg at 8 p.m. for two weeks, with the aim of maintaining F VIII level > 50%. Every 48 h, before the administration of concentrate of FVIII, blood was taken to check Hb, PTT and FVIII. The variations of FVIII biological activity levels during the hospital stay are summarised in Figure 3. Antibiotic intravenous post-operative prophylaxis with Vancomycin 500 mg every 12 h was continued until surgical drain removal. The surgical drain was kept for 24 h and then removed with a residual volume of 300 mL of serosanguinous liquid.

The patient was transfused with one RBC unit the day-4, the day-6 and the day-7 after surgery owing to post-operative anaemia. A 21-days hospital stay was necessary in order to achieve a good clinical condition.

**Figure 4:**
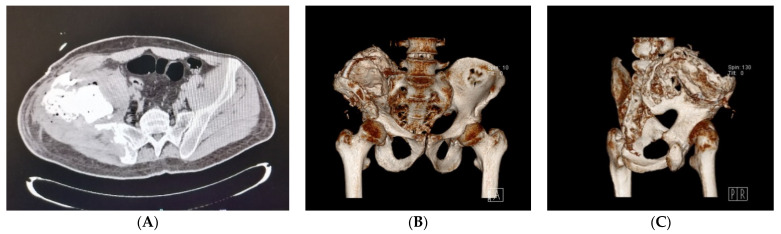
Postoperative CT scan (**A**) and 3D reconstruction (**B**,**C**) of the pelvis.

In the immediate post-operative period, X-ray and CT of the pelvis showed emptying of the pseudotumour and correct positioning of screws and metal cerclage (Figure 4). Physiotherapy was started during hospitalisation, from day-1 after surgery and continued during the hospital stay. The patient was discharged at home with the indications for the continuation of physiotherapy, with cautious and progressive recovery of total flexion of the trunk and right thigh. Walking was allowed with aids and load pain-compatible. The patient continued replacement therapy with recombinant F VIII and analgesic therapy with paracetamol. At discharge blood exams showed Hb 8.4 g/dL, aPTT 30.7 s, FVIII 96.5%, CRP 1.3 mg/dL, ESR 109 mm/h (<10). During the rehabilitation period the patient prophylaxis scheme was pd-FVIII 30 IU/Kg every other day. At the first outpatient visit, 8 days after discharge, the patient was in good general condition, he walked with aids, and the pain was well-controlled. The stitches were removed and medicated, no signs of inflammation were noted. Pelvis X-ray unchanged compared to the post-operative one. One month later the patient went to another emergency room for leakage of blood serum material from the surgical scar. A positive culture swab was found for MSSA. After two days, the patient was re-evaluated at our clinic, a wound dehiscence was observed, thus indication for wound revision was given. Five days later, the patient had a feverish episode (T 42 °C) with shaking chills and PCR increase. A broad-spectrum antibiotic therapy was begun and pd-FVIII 3000 IU at 8 and 1000 U at 20 was continued. Therefore, the patient was hospitalised at our Institution for wound surgical revision. Blood exams at the entrance showed Hb 10.6 g/dL, leukocytes 5.37 × 10^3^/uL (4.00–10.00), CRP 0.97 mg/dL, ESR 85 mm/h. Preoperatively, pelvic CT was performed, and it showed a voluminous collection of infected blood fluids both on the pelvic side along the planes of the ileo-psoas muscle and on the buttock side. The fluid collection extended to the paravertebral musculature, the quadrate loin muscle, and the external oblique muscle superiorly and posteriorly. In the postero-superior site, the collection went beyond the muscle fascia and extended into the subcutaneous fat layer. Therefore, surgical toilet and abundant irrigation was performed. During the surgical procedure, swabs were carried out for culture tests, later found negative for infection. The patient was discharged with a recommendation to continue antibiotic therapy with Rifampicin and Levofloxacin for 5 days and to continue the factor VIII replacement therapy. In this second hospitalisation a 11-days hospital stay was necessary in order to achieve optimal medical condition.

**Figure 5:**
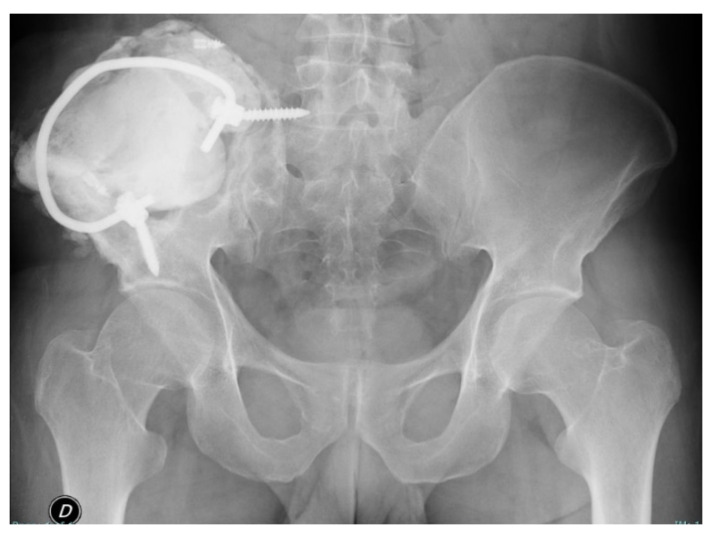
One-month post-operative radiograph control.

Patients were followed at outpatient clinic, at 7, 14 and 21 days. He was always in good general condition with surgical wound healed without signs of inflammation. Right hip ROM was almost complete at one month with control X-ray (Figure 5) unchanged than the post-operative one. At the 6- and 12-month outpatient visit the patient was in good clinical conditions, he denied disorders or functional limitations in every-day life. Currently, the patient is checked with an X-ray every 6 months.

The patient continued prophylaxis with pd-FVIII 30 IU/Kg three times a week. No further bleeding manifestations occurred.

## 3. Discussion

Pseudotumour is an uncommon but potentially severe debilitating complication of bleeding disorders like haemophilia. Pseudotumour is a chronic encapsulated haematoma that is not resorbed and continues to expand due to recurrent haemorrhages [6] developing a tumour-like form: capsulated masses containing coagulated blood and necrotic tissue [2]. The bleeding can arise spontaneously or, as in the case here reported, as a consequence of trauma.

Gilbert distinguished two types of pseudotumour based on location; the proximal type, seen most commonly in adults, occurs in the pelvis (especially in the ilium), and long bones extremities; in children there is a predilection for distal pseudotumours that occur in the small bones of the hands and feet [3].

Regarding the involvement of soft tissues, pseudotumours mainly affect those of the thigh, the gluteal region and the iliopsoas muscle, with possible simultaneous bone involvement.

Muscular pseudotumours can be classified as intra- or extra-muscular. Usually, intramuscular lesions remain localised, although they can sometimes expand towards the bone component. Pelvic haemophilic pseudotumours that develop after bleeding into the psoas or illiacus muscles may grow quite large and cause significant erosion of adjacent bone [7], like in our case.

Unfortunately, most of the haemophilic pseudotumours remain painless and asymptomatic, until they enlarge and compress adjacent structures (mass effect) causing pathological fractures, skin and soft tissue necrosis, fistula or infection [8,9].

In addition to the clinic, instrumental examinations such as X-ray, CT, MRI, ultrasound can be used for the diagnosis. On radiographs, intraosseus pseudotumours appear as well defined, uni- or multilocular, expansile lytic lesion of variable size with geographic pattern of bone destruction; a CT scan may differentiate the pseudotumours from the other tumours [10]; MRI is especially helpful for detecting intramedullary and soft tissue changes, such as recent bleeding and clots, but also for follow-up.

One limitation of MRI is its inability to allow differentiation between acute and subacute hematomas and abscesses [3].

There is no consensus on the management of haemophilic pseudotumour. Modalities for treatment include replacement therapy, local irradiation alone or combined with factor replacement, surgery and conservative measures such as percutaneous drainage and embolisation [3,11].

In general, the risk of pathological fracture, intractable pain, skin fistula or infection, huge mass with neurovascular compression had been the indication to implement surgical management first [2,12,13,14,15].

The best treatment for pseudotumours in patients with congenital coagulopathies is prevention [2], that is, proper long-term haematological treatment of muscle hematomas, until total resolution (reabsorption) confirmed by imaging studies—ultrasonography, CT or MRI [2].

As a general principle, mild-to-moderate lesions of recent origin, not causing pain or deformity can be managed with deficient factor infusions and immobilisation [16,17,18,19]. Immobilisation and replacement therapy for at least 8 weeks leads to healing in most patients [19]. Generally, factor replacement therapy, patient-tailored and well balanced with thromboprophylaxis, is a fundamental component of therapy regardless of any other interventions used [20,21]. As is stated in our case, continuous factor VIII replacement therapy was proven effective in preventing bleeding complications during the operation and the prolonged hospitalisation. There are several studies in literature in favour of surgery in patients with haemophilia A combined with continuous infusion or boluses of factor VIII to decrease risk of haemorrhage [11].

Therapeutic arterial embolisation had been used in the management of large haemophilic tumours, especially of the pelvic region [22]. However, the effect is temporary and it better serves as an adjunctive procedure, shrinking the lesion to make it more amenable to surgical resection [22,23]. Our experience confirmed the role of arterial embolisation in the bleeding control in the intra and peri-operative period.

Surgery is generally curative and it is cited as the most effective treatment for pseudotumour [16,23,24].

However, due to localisation, size and other factors, not all pseudotumours are amenable to surgery [22].

Surgical excision is indicated for large lesions, imminent rupture of pseudotumour, chronic pseudotumours or failure of conservative measures [16,17,19,25,26,27]. Soft-tissue pseudotumours may require excision to prevent skin necrosis, if there is evidence of nerve compression or if a tissue diagnosis is required to rule out neoplasm [17,18].

Surgical management of pseudotumour involving pelvis bone was previously described [7,10,11,28,29].

Pennekamp et al. [29] reported successful giant right iliac wing pseudotumour excision associated to a compound osteosynthesis with 20-hole reconstruction plate (attached to the remnants of the posterior superior iliac spine and the anterior acetabulum with screws and 3.0-mm), K-wires and bone cement to provide containment for the lesser pelvis and to fill dead space after resection of the destroyed ilium; the complication reported was the migration of a K-wire that required surgical revision because of the risk of rectal perforation.

In our case, too, pelvis containment was needed, and it was realised by adopting a novel surgical technique which involves the use of metal bar, fixing screws, polyglycolic acid MESH and bone cement in order to build up an artificial ilium-like bony mass. The choice of using fixing screws and a bar was dictated by the necessity of minimise the risk of implant mobilisation.

To our knowledge this is the first time that a PSRS has been used for the surgical management of a haemophilic pseudotumour.

PSRS with various techniques have been proposed for pelvic ring reconstruction following pelvis malignant tumour resection [30,31,32]. Functional reconstruction with the PSRS is described as a reliable and effective procedure, particularly seen in pelvic resection type I and IV [33,34].

A surgical MESH is a sterile woven material designed for permanent implantation within the body during open or laparoscopic procedures. A wide range of mesh implants is available with two main functions: to stabilise and strengthen soft tissue defects and to act as a sling to support prolapsed organs and viscera [35]. In our case two polyglycolic acid MESH were employed to contain the bone cement in order to reconstruct an artificial ilium-like bony mass avoiding leaving a dangerous dead space.

PMMA, commonly known as bone cement, has proved to be one of the most versatile and lasting biomaterials in orthopaedics having an important role in joint replacement, as well as spinal and tumour surgery [36,37]. At present, PMMA is still the most commonly used filling material in kyphoplasty and vertebroplasty [38]. Bone cement also plays a vital role in the treatment of giant cell tumours of bone, where it has been used since 1969 as a packing material after a curettage procedure [39,40]. However, although used universally for many years, PMMA bone cement is not without its problems. PMMA cement may provide instant stability, can grant immediate mechanical strength and early recovery of weight bearing [41] but is not a biologically integrated bone filler [42]. In addition, it is neither osteoinductive nor osteoconductive and does not remodel [39]. Moreover, bone cement implantation syndrome (BCIS) and thermal injury risk during implantation are other well-known complications related to the use of bone cement [39].

Surgery is curative but inherently risky, therefore a careful patients selection is mandatory [22]. Surgical risks include: catastrophic haemorrhage; incapacitating surgery, such as limb amputation; infection; fistula formation and vascular and neurological damages [19,22,43].

Treatment is especially challenging in patients with large masses and extensive bone destruction [22].

Infection remains a frequent complication of reconstruction with haemipelvic allografts or autoclaved grafts and prosthesis after internal hemipelvectomy, with reported rates varying from 12% to 47%. The following factors contribute to the high risk of infection: the prolonged surgical time necessary to resect pelvic tumours, the proximity of the rectum and genitourinary tract, the large dead space after pelvic bone resection and the large amount of foreign material implanted in the surgical wound [34,44]. In our case many of the aforementioned risk factors were present in addition to the skin fistula. Therefore, to decrease infection risk, we adopted peri-operative and post-operative antibiotic prophylaxis with vancomycin until the surgical drain removal, early performed at 24 h post-surgery. Moreover, antibiotic-coated bone cement was employed for both structural and filler purpose in order to eliminate the post excision dead space.

According to the review of Austin et al. [45] surgical excision, associated with various composite fillings, remains the most common management strategy. The same work has highlighted the occurrence of post-operative complications in 38% of the cases, including infections (13.9%), fistula formations (13.9%), poor wound closure (10.1%), haemorrhages (8.9%) and mortality (3.8%) [45].

In our case the infection was controlled by antibiotic therapy and the recidivism fistula surgical revision with fistulectomy was successful.

## 4. Conclusions

Although there is still no consensus regarding the therapeutic management of pseudotumours, the favourable course of the case described above allows us to suggest eradicating surgery as the first line of treatment, especially in the case of large and long-lasting lesions associated with complications, e.g., infection and fistula. However, in order to have the best results in the long term, a not only surgical, but interdisciplinary approach is necessary.

Therefore, early diagnosis and a variety of treatment modality should be initiated to prevent the haemophilic pseudotumours progressing to intractable status. A multicentre study or nationwide population-based study should be performed to explore the treatment efficacy in the future.
